# Inotropic interventions do not change the resting state of myosin motors during cardiac diastole

**DOI:** 10.1085/jgp.201812196

**Published:** 2019-01-07

**Authors:** Marco Caremani, Francesca Pinzauti, Joseph D. Powers, Serena Governali, Theyencheri Narayanan, Ger J.M. Stienen, Massimo Reconditi, Marco Linari, Vincenzo Lombardi, Gabriella Piazzesi

**Affiliations:** 1 PhysioLab, University of Florence, Firenze, Italy; 2 European Synchrotron Radiation Facility, Grenoble, France; 3 Department of Physiology, VU University Medical Center, Amsterdam, The Netherlands

## Abstract

When striated (skeletal and cardiac) muscle is in its relaxed state, myosin motors are packed in helical tracks on the surface of the thick filament, folded toward the center of the sarcomere, and unable to bind actin or hydrolyze ATP (OFF state). This raises the question of whatthe mechanism is that integrates the Ca^2+^-dependent thin filament activation, making myosin heads available for interaction with actin. Here we test the interdependency of the thin and thick filament regulatory mechanisms in intact trabeculae from the rat heart. We record the x-ray diffraction signals that mark the state of the thick filament during inotropic interventions (increase in sarcomere length from 1.95 to 2.25 µm and addition of 10^−7^ M isoprenaline), which potentiate the twitch force developed by an electrically paced trabecula by up to twofold. During diastole, none of the signals related to the OFF state of the thick filament are significantly affected by these interventions, except the intensity of both myosin-binding protein C– and troponin-related meridional reflections, which reduce by 20% in the presence of isoprenaline. These results indicate that recruitment of myosin motors from their OFF state occurs independently and downstream from thin filament activation. This is in agreement with the recently discovered mechanism based on thick filament mechanosensing in which the number of motors available for interaction with actin rapidly adapts to the stress on the thick filament and thus to the loading conditions of the contraction. The gain of this positive feedback may be modulated by both sarcomere length and the degree of phosphorylation of myosin-binding protein C.

## Abstract (precis)

Thick filament mechanosensing has been proposed as the mechanism by which myosin motors in cardiac muscle become available to bind actin. Accordingly, Caremani et al., using x-ray diffraction from intact rat trabeculae, show that myosin motors fully return to their OFF state during diastole independently of inotropic interventions.

## Introduction

The generation of force and shortening in striated muscle (skeletal and cardiac) is due to the cyclic ATP-driven interactions of myosin motors emerging from the thick filament with the neighboring thin, actin-containing filaments. The mechanical performance of muscle is under the control of both thin and thick filament regulation. The start signal is the increase of intracellular Ca^2+^ concentration ([Ca^2+^]_i_), induced by cell membrane depolarization by the action potential, followed by Ca^2+^ binding to troponin in the thin filament and structural changes in the regulatory troponin–tropomyosin complex that release the actin sites for binding of myosin motors (Ebashi et al., 1969; Huxley, 1973; Gordon et al., 2000). A second regulatory mechanism, based on thick filament mechanosensing (Linari et al., 2015; Reconditi et al., 2017), recruits myosin motors from their OFF state in which they lie along the surface of the thick filament, folded toward the center of the sarcomere, unable to bind actin (Woodhead et al., 2005; Zoghbi et al., 2008) or hydrolyze ATP (Stewart et al., 2010).

In contrast to skeletal muscle, in which the thin filament is kept activated by the maintained high level of [Ca^2+^]_i_ induced by repetitive firing of action potentials, in the heart the mechanical activity (systole) consists of short periodic contractions (twitches) triggered by single action potentials. During systole, the blood is pumped by ventricles into the arterial circulation. In the resting period between two systoles (diastole), the heart is filled by the blood from the venous return. In contrast to skeletal muscle, cardiac muscle [Ca^2+^]_i_ may not reach the level for full thin filament activation during systole. Consequently, the mechanical response depends on both [Ca^2+^]_i_ and the Ca^2+^ sensitivity of the filament (Allen and Kentish, 1985; ter Keurs, 2012), parameters that are under the control of several regulatory systems, either intrinsic, like the relation between sarcomere length (SL) and systolic force (a property known as length-dependent activation [LDA]; Sagawa et al., 1988; de Tombe et al., 2010), or extrinsic, like neuro-humoral control of the degree of phosphorylation of several sarcomere proteins: among them the regulatory light chain (RLC) of the myosin itself, the regulatory protein troponin I (TnI) on the thin filament, myosin-binding protein C (MyBP-C) on the thick filament, and the cytoskeletal protein titin (Herron et al., 2001; Hidalgo and Granzier, 2013; Kumar et al., 2015; Kampourakis et al., 2016). LDA is the cellular basis of the Frank–Starling law of the heart that, in its classical formulation, relates the pressure exerted on the blood during the contraction of the ventricle (end-systolic pressure) to its filling during the relaxation (end-diastolic volume), in this way ensuring the dynamic equilibrium between the two circulatory systems (pulmonary and systemic) driven by two pumps in series.

LDA is the result of a chain of not yet defined events relating a mechanosensor of the SL to the number of force-generating motors, and consequently to the force (Spudich, 2014; Caremani et al., 2016), through an integrated control of the degree of activation of the thin and thick filaments. An important role in this regulatory mechanism is played by MyBP-C, which is bound with its C terminus to the backbone of the thick filament in the central one third of the half sarcomere (C zone) and extends from the thick filament to establish, with its N terminus, dynamic interactions, controlled by the PKA-dependent degree of phosphorylation, with either the actin filament or the rod-like S2 domain of the myosin (Moos et al., 1978; Rybakova et al., 2011; Pfuhl and Gautel, 2012).

Relevant mechanical and structural features toward the clarification of the role of MyBP-C in dual filament regulation were obtained using demembranated cardiac myocytes and selecting conditions to modulate phosphorylation of MyBP-C without the influence of the other PKA target proteins in the sarcomere involved in either the Ca^2+^ handling (L-type Ca^2+^ channels in the cell membrane, ryanodine receptors, and phospholamban) or thin filament regulation (TnI). It was found that dephosphorylated MyBP-C preferentially binds myosin, reducing the probability of myosin to bind actin. This inhibition is relieved by MyBP-C phosphorylation, or by MyBP-C gene ablation (Harris et al., 2002), leading, in particular at intermediate [Ca^2+^], to a higher rate of force development and a larger power output (Herron et al., 2001; Korte et al., 2003). The structural counterpart, investigated with x-ray diffraction (Colson et al., 2010, 2012), is the finding that MyBP-C phosphorylation of skinned mouse trabeculae in relaxing solution induces an increase in the intensity of the 1,1 equatorial reflection (I_1,1_; generated by the lattice planes formed by the actin and myosin filaments) relative to the intensity of the 1,0 equatorial reflection (I_1,0_; generated by the lattice planes formed only by myosin filaments). This result is an indication of the movement of the mass density constituted by the myosin heads from the proximity of the thick filament toward the actin filament, and presumably of an increased probability for myosin to bind actin once Ca^2+^ switches on the thin filament. MyBP-C phosphorylation-dependent mobilization of the myosin heads has been recently confirmed by EM on isolated thick filament (Kensler et al., 2017). In this respect, MyBP-C appears to play a role in the intermolecular–intramolecular interactions by determining the helical packing of myosin heads along the thick filament in the OFF state. In fact, these interactions are generated not only within the myosin molecules (head–head and head–tail interactions responsible for what is called the interacting head motif [IHM]; Alamo et al., 2008), but also with other thick filament proteins such as MyBP-C and titin that are assembled so as to match the 43-nm helical periodicity of the myosin molecules (Rome et al., 1973; Labeit et al., 1992). Titin, which spans the whole half sarcomere, connecting the Z-line at the end of the sarcomere with the tip of the myosin filament and then running, bound to the surface of the thick filament, up to the M-line at the center of the sarcomere, could play a role in LDA, as it is able to transmit the stress to thick filament also in the resting sarcomere, when no motors are attached to actin. Indeed, modulation in titin-dependent passive force either by stretch (Cazorla et al., 2001) or by engineered changes in titin stiffness (Fukuda et al., 2010, and references therein) alters Ca^2+^ sensitivity of force in skinned cardiac myocytes.

A still not defined interplay among these proteins is likely to be the structural basis for the potentiation of cardiac contraction (positive inotropic effect) by both increased SL and increased degree of MyBP-C phosphorylation in the diastole preceding the contraction. This idea is sustained by the effects of MyBP-C phosphorylation or ablation on the length dependence of the Ca^2+^ sensitivity (Kampourakis et al., 2014; Mamidi et al., 2014, 2016) and by length-dependent structural changes recorded with fluorescent probes in thin and thick filaments (Zhang et al., 2017). However, the idea does not find clear support when these inotropic interventions are investigated on the cardiac preparation of choice, the intact trabecula from the rat ventricle, in which it is possible to apply high-resolution mechanical methods and define the mechanokinetic features of the myosin motors (Caremani et al., 2016; Pinzauti et al., 2018). The most relevant result, obtained by combining sarcomere-level mechanics and x-ray diffraction in intact trabeculae, is the finding that, independent of the diastolic SL, the degree of thick filament activation is under the control of the load during systole via a rapid positive feedback between the stress on the filament and the switching ON of myosin motors (Reconditi et al., 2017; Piazzesi et al., 2018). This result suggests that the control of thick filament activation is downstream from Ca^2+^-dependent thin filament activation. Data in the literature referring to signals that should represent the structural basis of LDA in diastole, such as the movement of myosin motors away from the thick filament, are contradictory: the intensity of the third-order meridional reflection (M3), originating from the ∼14.5-nm axial repeat of the myosin heads, has been found to increase with SL, and this increase has been interpreted as an increase of the ordering of the heads more perpendicular to the filament axis (Farman et al., 2011); more recently, however, the same group reported that, based on the absence of a significant increase in both the intensity of M3 reflection and the intensity ratio between I_1,1_ and I_1,0_, there is no evidence for a change in the radial position of the heads in response to a stretch of the trabecula in diastole, while changes in the intensity of other myosin- and actin-based reflections would indicate some stretch-induced increase in mass ordering in both filaments (Ait-Mou et al., 2016). Indeed, these authors find that I_1,1_/I_1,0_ decreases upon stretch, a result that is not expected given the evidence from demembranated cardiac cells that, even at low [Ca^2+^], inotropic interventions such as an increase in either SL (Kampourakis et al., 2016; Zhang et al., 2017) or the degree of phosphorylation of MyBP-C (Colson et al., 2012) imply a mobilization of the myosin heads from their OFF state accompanied by a shift of mass toward the actin filament.

Here we report an investigation of the effects on the regulatory state of the thick filament in diastole of the two above-mentioned inotropic interventions, namely an increase in SL and the PKA-induced increase of phosphorylation of sarcomeric proteins, including MyBP-C, by addition to the solution of the β-adrenergic effector isoprenaline (ISO). For this, we exploited the nanometer-micrometer scale x-ray diffraction possible at beamline ID02 of the European Synchrotron Radiation Facility (ESRF; Grenoble, France) to record the structural changes undergone by the thick filament during the diastole of electrically paced intact trabeculae from rat ventricle either by increasing SLs in the range 1.95–2.25 µm or following the addition of ISO (10^−7^ M). With 1 mM Ca^2+^ in the bathing solution ([Ca^2+^]_o_), both inotropic interventions are able to almost double the twitch peak force (*T*_P_). The 2-D patterns collected at ESRF allow a comparative analysis of the changes of the intensity of the first myosin layer line (ML1) and of the intensity, spacing, and fine structure of the meridional reflections up to the sixth order (M1–M6). All these reflections originate from the three-stranded quasi-helical symmetry with 43-nm axial periodicity followed by the myosin heads when they lie on the surface of the thick filament in their resting (OFF) state. The analysis is done also on the first-order troponin meridional reflection (T1), originating from the axial periodicity of troponin complex on the thin filament, and 1,0 and 1,1 equatorial reflections, originating from the lattice planes formed by the double hexagonal symmetry of actin and myosin filaments in the transversal section of the myocyte. Apart from a 20% drop in the intensity of both MyBP-C– and troponin-related meridional reflections upon addition of ISO, none of the signals marking the state of the thick filament in diastole was significantly affected by either intervention, indicating that, in agreement with the mechanosensing mechanism controlling the switching ON of the myosin motors, thick filament activation occurs independently of thin filament activation and downstream with respect to it.

## Materials and methods

### Animals and ethical approval

Male rats (*Rattus norvegicus*, strain Wistar Han; 230–280 g; Charles River Laboratories) were housed at the Centro di Stabulazione Animali da Laboratorio, University of Florence, Firenze, Italy, and at the Bio-Medical Facility (ID17) of the ESRF, under controlled temperature (20 ± 1°C), humidity (55 ± 10%), and illumination (lights on for 12 h daily, from 7 a.m. to 7 p.m.). Food and water were provided ad libitum. All animals were treated in accordance with both the Italian regulation on animal experimentation (authorization no. 956/2015 PR) in compliance with Decreto Legislativo 26/2014 and the European Union regulation (directive 2010/63). On the day of the experiment, the rat was anesthetized with isoflurane [5% (vol/vol)] and, as soon as the animal attained a state of deep anesthesia, as judged by the absence of the pedal reflex and the loss of the muscle tone in the hind limb, the heart was rapidly excised, placed in a dissection dish, and retrogradely perfused with a modified Krebs–Henseleit solution (composition in mM: NaCl, 115; KCl, 4.7; MgSO_4_, 1.2; KH_2_PO_4_, 1.2; NaHCO_3_, 25; CaCl_2_, 0.5; and glucose, 10), containing 20 mM 2,3-butanedione monoxime, and equilibrated with carbogen (95% O_2_, 5% CO_2_, pH 7.4).

### Sample preparation

A thin, unbranched trabecula was dissected from the right ventricle under a stereomicroscope. The trabecula was set at the length (*L*_t_) at which it was just taut, and its width (*w*) and thickness (*h*) were measured using an eyepiece with a graduated scale. The cross-sectional area (CSA) was calculated as *w* × *h* × π/4. The trabecula was then transferred into a temperature-controlled trough (1.2-ml volume) perfused at 1.2 ml/min with oxygenated Krebs–Henseleit solution (27°C) and attached, via titanium double hooks anchored to aluminum strips clamping the extremities, to the lever arms of a strain gauge force transducer (valve side) and a loudspeaker motor (wall side) for mechanical measurements. The characteristics of the force and length transducers and the procedure of attachment of the trabecula to the levers have been previously described (Lombardi and Piazzesi, 1990; Caremani et al., 2016). The SL was set at ∼2.1 µm at rest by using a 40× dry objective and a 25× eyepiece, and the corresponding trabecula length (*L*_0_) was measured again and the CSA corrected for the change in length (*L*_0_ − *L*_t_), assuming constant volume behavior. The dimensions of the preparations used for the mechanical experiments done at PhysioLab, University of Florence, were (mean ± SD, *n* = 4) *w*, 240 ± 100 µm; *h*, 74 ± 20 µm; CSA, 14,300 ± 8,700 µm^2^; and *L*_0_, 2.84 ± 0.46 mm. Only trabeculae of CSA > 12,000 µm^2^ were used at ESRF, to have an adequate signal-to-noise ratio in the x-ray signals (dimensions, *w*, 410 ± 170 µm; *h*, 100 ± 30 µm; CSA, 31,500 ± 11,900 µm^2^; and *L*_0_, 2.49 ± 0.31, *n* = 4).

#### Experimental protocols

##### Mechanical measurements

**Figure 1.**
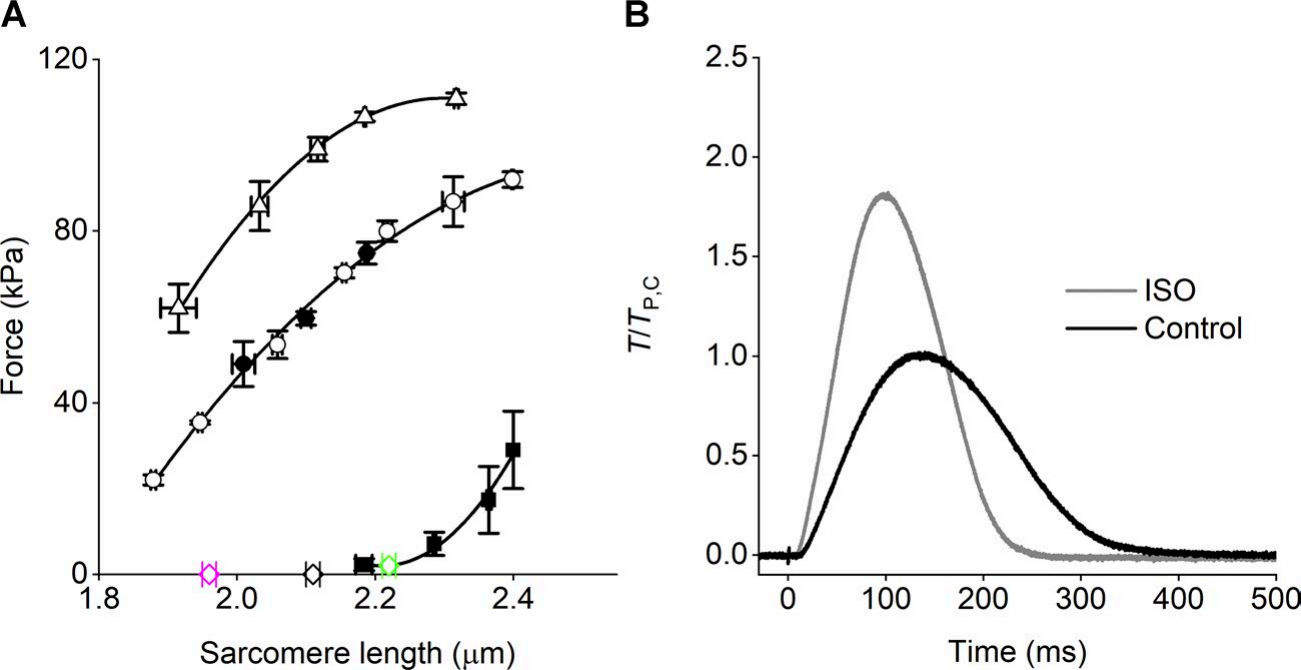
****Mechanical responses under the two inotropic protocols. (A)**** Relations between *T*_P_ and SL at 1 mM [Ca^2+^]_o_ (circles) and 2.5 mM [Ca^2+^]_o_ (triangles). Passive force shown by squares. The lines are polynomial fits to points. Data from Caremani et al. (2016). The force–SL relation is unique independent of fixed-end (filled circles) or sarcomere-length clamp condition (open circles). Open diamonds indicate the three SL used for the x-ray study of the effect of SL with [Ca^2+^]_o_ 1 mM (error bars are SEM; four trabeculae). **(B)** Time course of the twitch force with [Ca^2+^]_o_ 1 mM and SL 2.1 µm either in control (black trace) or in the presence of 10^−7^ M ISO (gray trace). Force is relative to *T*_P_ in control. The artifact on the force trace marks the stimulus start. Length of the trabecula, 2.7 mm; CSA, 43,200 µm^2^; temperature, 27.1°C.

Intact trabeculae were electrically stimulated by means of two platinum plate electrodes, 4 mm apart, with bipolar pulses of 0.5-ms duration and amplitude 1.5 times the threshold voltage. Measurements were made at the steady state of the contraction–relaxation cycle during electrical pacing at 0.5 Hz. A striation follower (Huxley et al., 1981) was used to record SL changes in a 0.7–1.5-mm-long segment selected along the central region of the preparation. The two inotropic interventions studied in this work, the increase in SL and the addition of the β-adrenergic agonist ISO were defined in order to almost double the peak twitch force (*T*_P_) of the trabecula electrically paced at 0.5 Hz. The first protocol, which was mechanically characterized in previous work (Caremani et al., 2016 and references therein), exploits the SL dependence of *T*_P_ with [Ca^2+^]_o_ 1 mM, which provides that at ∼2.2 µm *T*_P_ is twice the value at ∼1.95 µm (Fig. 1 A and Table 1). The second protocol has been selected by preliminarily titrating the effect of ISO on *T*_P_ under our experimental conditions—that is, with [Ca^2+^]_o_ 1 mM and SL ∼2.1 µm. ISO increases *T*_P_ up to a maximum of twice *T*_P_ in control (*T*_P,C_) in a dose-dependent manner with pISO_50_ = 8.6 (corresponding to 2.5 × 10^−9^ M ISO). In the experiments described in this paper, a saturating concentration of 10^−7^ M ISO has been used, which provided an increase in *T*_P_ of 71% (Fig. 1 B and Table 1).

**Table 1.** Increase of the *T*_P_ relative to control (*T*_P,C_) following two inotropic interventions with [Ca^2+^]_0_ 1 mM

**SL (µm)**	**A**	**B**
1.95 ± 0.01	0.57 ± 0.01 (6)	
2.11 ± 0.01	1	
2.22 ± 0.01	1.27 ± 0.02 (7)	
2.10 ± 0.05		1.71 ± 0.09 (8)

**(A)** Increase of SL from 1.95 µm to 2.11 and 2.22 µm. **(B)** Addition of 10^−7^ M ISO. The number of trabeculae for each protocol are in parentheses. Data in A from Caremani et al. (2016); data in B from the four trabeculae used for the x-ray measurements plus four trabeculae used for the mechanical experiments.

##### Mechanical data collection and analysis

Force, motor lever position, and SL signals were recorded at sampling intervals of 100 µs with a multifunction input/output board (PXIE-6358; National Instruments). Dedicated computer programs written in LabVIEW (National Instruments) and Origin 2015 (OriginLab Corporation) were used for analysis.

##### X-ray diffraction data collection

Following SL adjustment at 2.1 µm (see above), the trough was sealed to prevent solution leakage and mounted with the trabecula axis vertical at beamline ID02 of the ESRF (Narayanan et al., 2018), which provided up to 2 × 10^13^ photons per second with 0.1-nm wavelength in a beam of size ∼300 µm (horizontal, full width at half maximum) and ∼50 µm (vertical) at the sample. The beam was attenuated for trabecula alignment. To minimize radiation damage, x-ray exposure was limited to the data collection period using a fast electromagnetic shutter (model LS500; nm; Laser Products, Inc.), and the trabecula was shifted along its axis by 100–200 µm between exposures. X-ray diffraction patterns were recorded using the FReLoN charge-coupled device–based detector with 2,048 × 2,048 pixels (50 × 50 mm^2^ active area). Pixels were binned by eight in the equatorial direction (perpendicular to the trabecula axis) before the readout to increase the signal-to-noise ratio.

**Figure 2.**
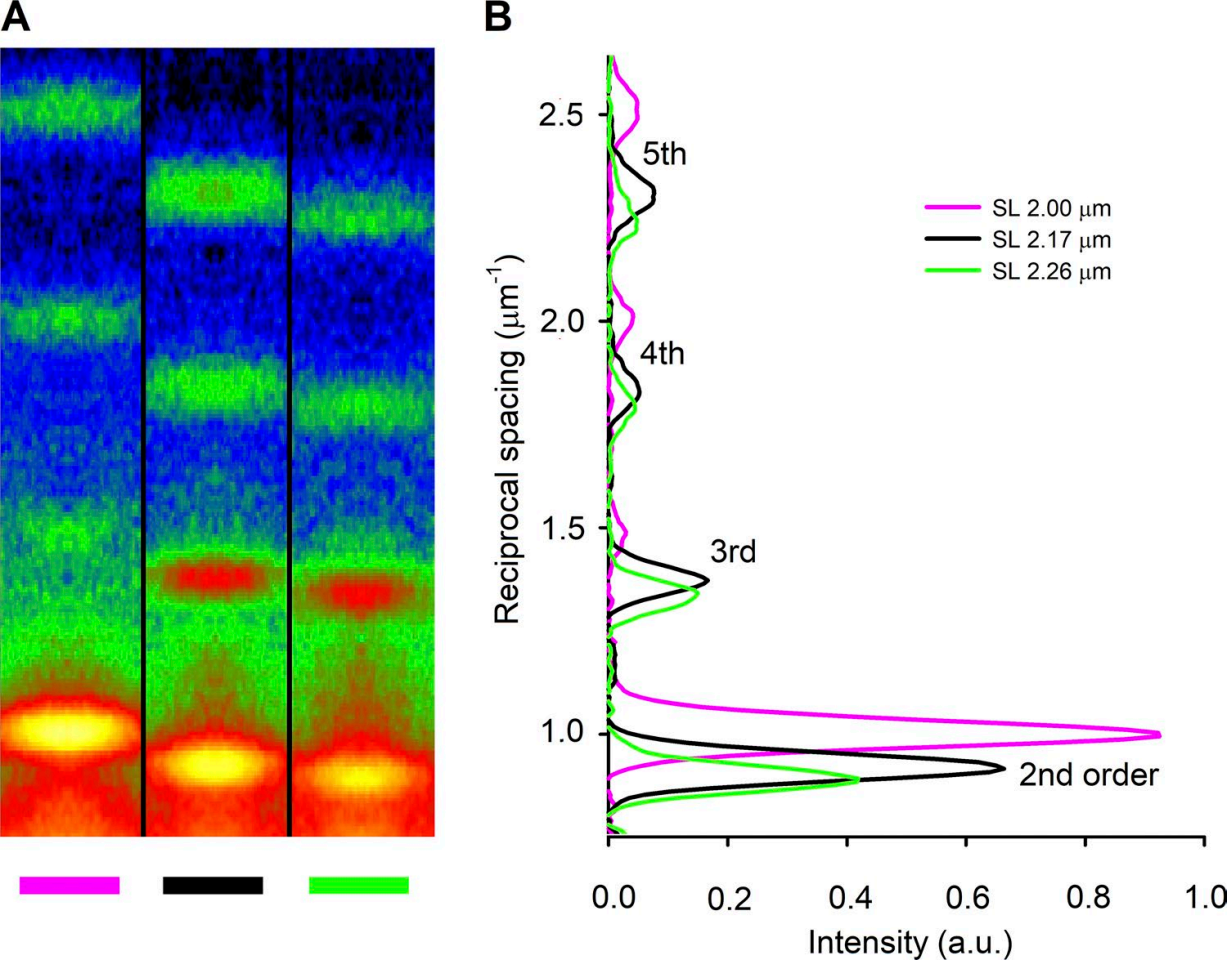
**X-ray sarcomere reflections recorded with the detector at 31 m from the preparation. (A)** Meridional slices of 2-D diffraction patterns from a trabecula in diastole at three different lengths as indicated by the color code (magenta, 2.81 mm; black, 3.19 mm; green, 3.40 mm; corresponding SLs indicated with the same color code in the inset in B), showing the orders of the sarcomere reflections from second to fifth. At this camera length, the first order is masked by the beam stop. Total exposure time, 1.8 ms for each pattern. **(B)** Meridional intensity profiles from axial integration of the 2-D patterns in A. The lines and the SLs reported in the inset have the same color code as the corresponding trabecula lengths in A. a.u., arbitrary units.

The x-ray detector was preliminarily placed at 31 m from the preparation to collect the sarcomere reflections (Fig. 2). To obtain a given SL, the trabecula length was first changed by the same percentage change estimated for the desired SL. Then the SL was measured by using the intense second-order sarcomere reflection, and, based on the difference between the measured and the desired SL, the trabecula length was adjusted again with an iterative procedure.

The detector was then moved to 1.6 m from the preparation, and x-ray patterns were recorded with 10-ms exposure windows in diastole just before the 0.5-Hz pacing stimulus. For each trabecula, the exposures were repeated three times at each of the three SLs, both in control and with ISO, with the order selected at random. In the four trabeculae used for the x-ray data analysis, the protocol was completed before a detectable sign of radiation damage appeared in the x-ray pattern. The number of trabeculae was chosen in order to measure intensities and spacings of the relevant x-ray reflections with adequate signal-to-noise ratio and minimize the number of animals to be sacrificed; this could be achieved with a small number of trabeculae because the x-ray signals can be measured with extremely high precision and low biological variability (Linari et al., 2000; Brunello et al., 2014; Reconditi et al., 2014).

##### X-ray data analysis

X-ray diffraction data were analyzed using Fit2D (A. Hammersley, ESRF), PeakFit (SYSTAT Software, Inc.), and IgorPro (WaveMetrix, Inc.). 2-D patterns were centered and aligned using the equatorial 1,0 reflection, and then quadrant folded horizontally and vertically. The distribution of diffracted intensity along the meridional axis of the x-ray pattern (parallel to the trabecula axis) was obtained by integrating the 2-D pattern from 0.009 nm^−1^ or 0.019 nm^−1^ on either side of the meridian, to optimize the signal-to-noise ratio for both the radially narrower (M1, M2, and T1) and wider (M3, M5, and M6) meridional reflections, respectively. Given the arcing of the reflections, to accurately determine the spacing, the narrower integration limits (±0.009 nm^−1^) were used for all the meridional reflections. The axial intensity distribution of the ML1 was obtained by integrating the region between 0.037 and 0.064 nm^−1^ from the meridional axis. Most background from the 1-D intensity distributions was removed using a convex hull algorithm; the small residual background was removed using the intensity from a nearby region of the 1-D intensity profile containing no reflections. The total intensities and the spacings of the reflections were then obtained by integrating the axial distribution in the corresponding regions and measuring their center of mass: M1, 0.0213–0.0247 nm^−1^; T1, 0.0249–0.0274 nm^−1^; M2, 0.0456–0.0469 nm^−1^; M3, 0.0676–0.0712 nm^−1^; M5, 0.115–0.117 nm^−1^; M6, 0.137–0.141 nm^−1^, and ML1, 0.0162–0.0238 nm^−1^. The limits for the ML1 integration were chosen to exclude the contribution of the partially overlapping first-order actin layer line. The intensity distribution of the low-angle reflections along the equator of the pattern was determined by integrating from 0.009 nm^−1^ on either side of the equatorial axis, and after background removal, the intensity and spacing of the equatorial reflections were determined with a simultaneous Gaussian fit on both 1,0 and 1,1 reflections in the equatorial profile. The intensities of all the reflections were then corrected to account for the different mass of the trabecula crossed by the x-ray beam at the different SL, which is inversely proportional to SL when the trabecula width is smaller than the horizontal beam size and inversely proportional to √SL in the opposite case.

## Results

**Figure 3.**
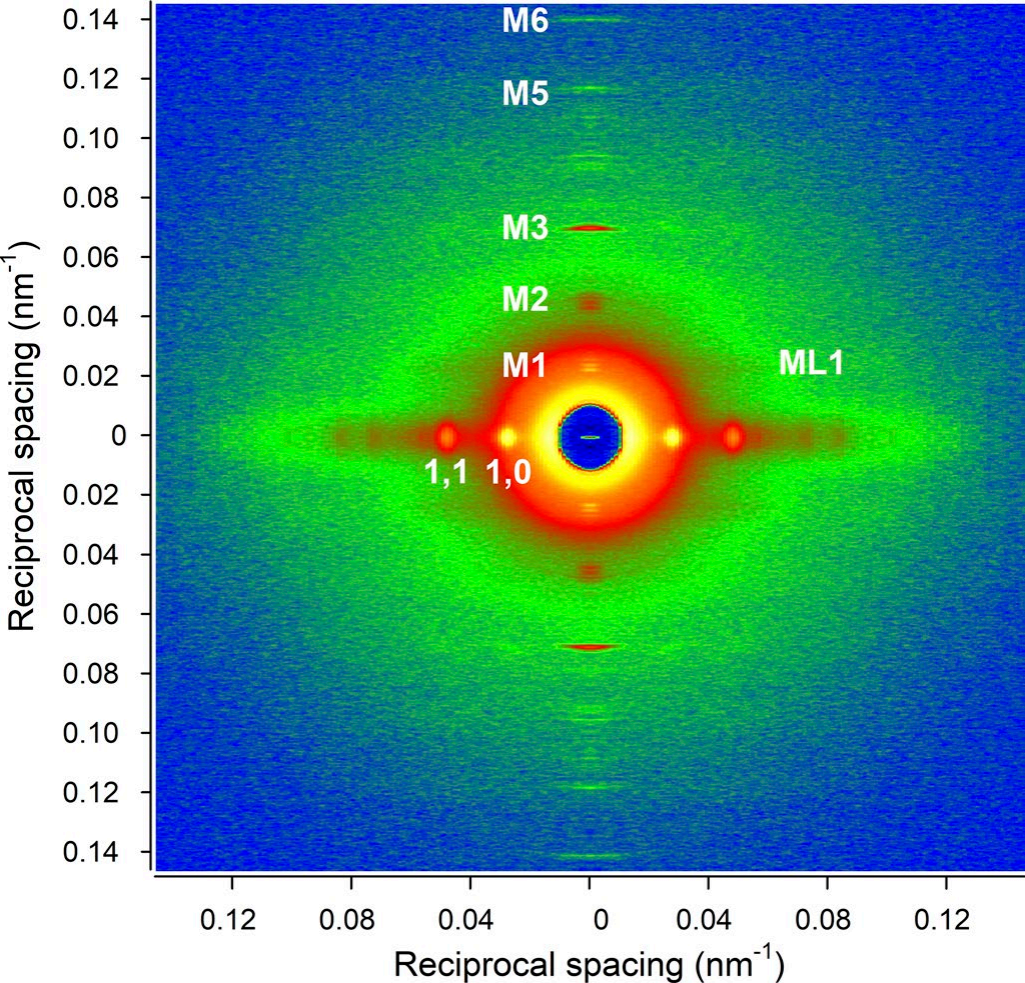
**X-ray diffraction pattern recorded from an intact trabecula in diastole with the detector at 1.6 m from the preparation.** The horizontal axis, perpendicular to the long axis of the trabecula, shows the low-angle equatorial reflections (1,0 and 1,1) from the lattice planes formed by the thick and thin filaments. The vertical axis, parallel to the trabecula axis, shows the meridional reflections from the axial periodicity of the proteins along the filaments up to the sixth order of the meridional myosin-based reflections (M1, M2, M3, M5, and M6; M4 is visible but not used in the x-ray analysis because of its complexity). The ML1 extends in the radial direction in correspondence to the M1 axial spacing. SL, 2.11 µm; total exposure time, 30 ms. The pattern has been quadrant folded to increase the signal-to-noise ratio.

The 2-D x-ray diffraction pattern recorded from a trabecula in diastole at 1.6 m from the preparation (Fig. 3) shows (a) the two intense low-angle equatorial reflections (1,0 and 1,1) along the horizontal axis (perpendicular to the trabecula axis), generated by the hexagonal array of filaments, (b) the ML1 at an axial spacing of 43 nm, due to the three-stranded quasi-helical symmetry of myosin molecules on the surface of the thick filament, and (c) up to the sixth order of the myosin-based meridional reflections (M1–M6) along the vertical axis (parallel to the trabecula axis) indexing on an axial periodicity of ∼43 nm. The spatial resolution achieved with vertically mounted trabeculae along the meridian is adequate to record the fine structure of the meridional reflections due to the x-ray interference between the two halves of the thick filament (Linari et al., 2000).

### Effects of the increase in SL on the x-ray pattern

**Figure 4.**
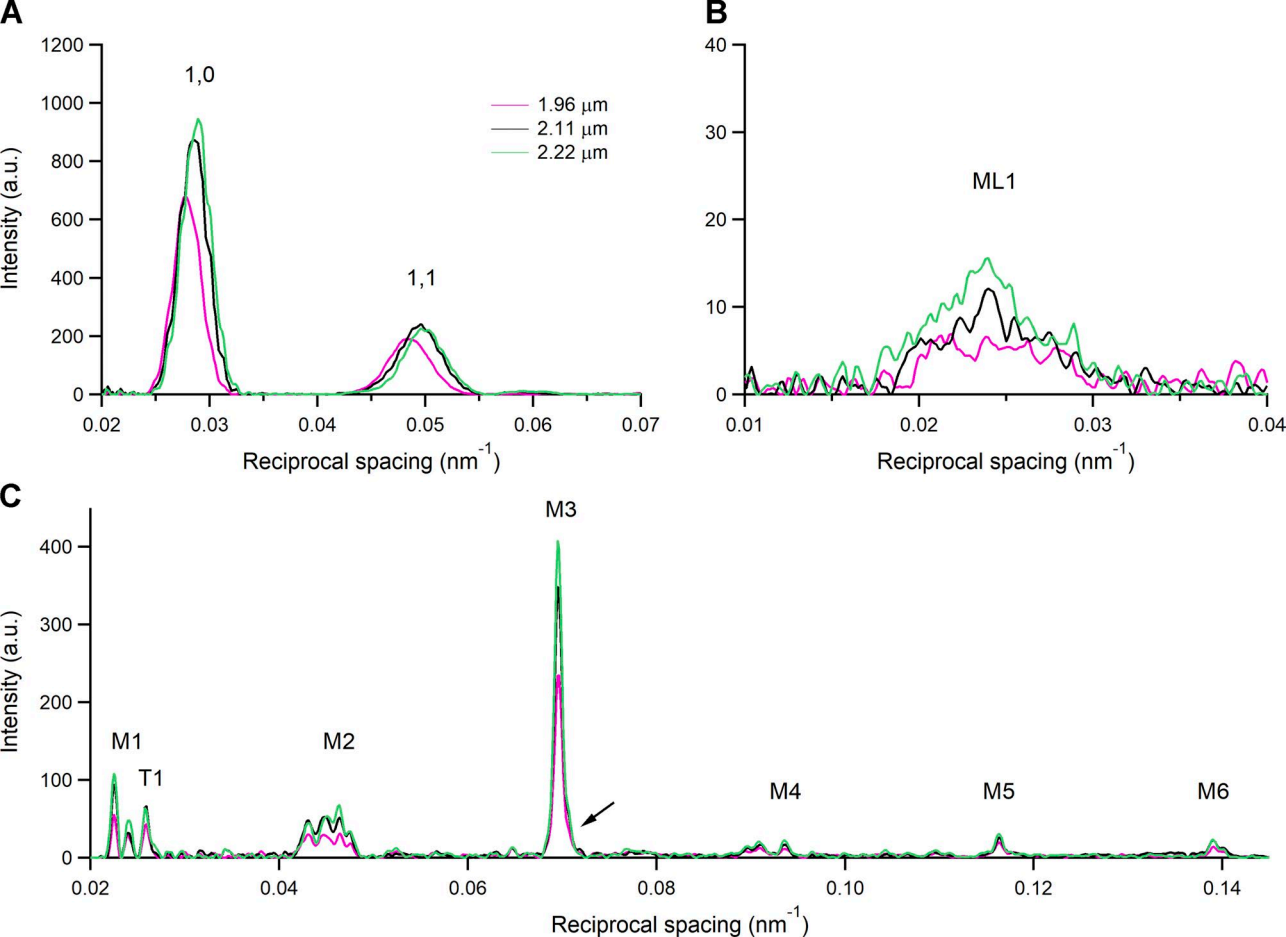
**1-D intensity distributions from x-ray diffraction patterns during the diastole at three different SLs. (A)** Intensity distribution along the equatorial axis with indicated 1,0 and 1,1. **(B)** Intensity distribution along the meridional direction of the ML1. **(C)** Meridional intensity distribution with indicated myosin-based reflections from M1 to M6 and the troponin-based T1 reflection. In the M2 cluster, only the third peak from the left, that corresponds to the second order of 43-nm M1 reflection, is used for the analysis (Reconditi et al., 2014; Ait-Mou et al., 2016). The arrow next to the M3 reflection indicates the small satellite on the high-angle side (for a higher resolution, see the expanded M3 profile in Fig. 8 E). Color code: magenta, SL 1.96 µm; black, SL 2.11 µm; green, SL 2.22 µm. Camera length 1.6 m, total exposure time for each SL, 120 ms, summed from the four trabeculae used in the x-ray experiments.

**Figure 5.**
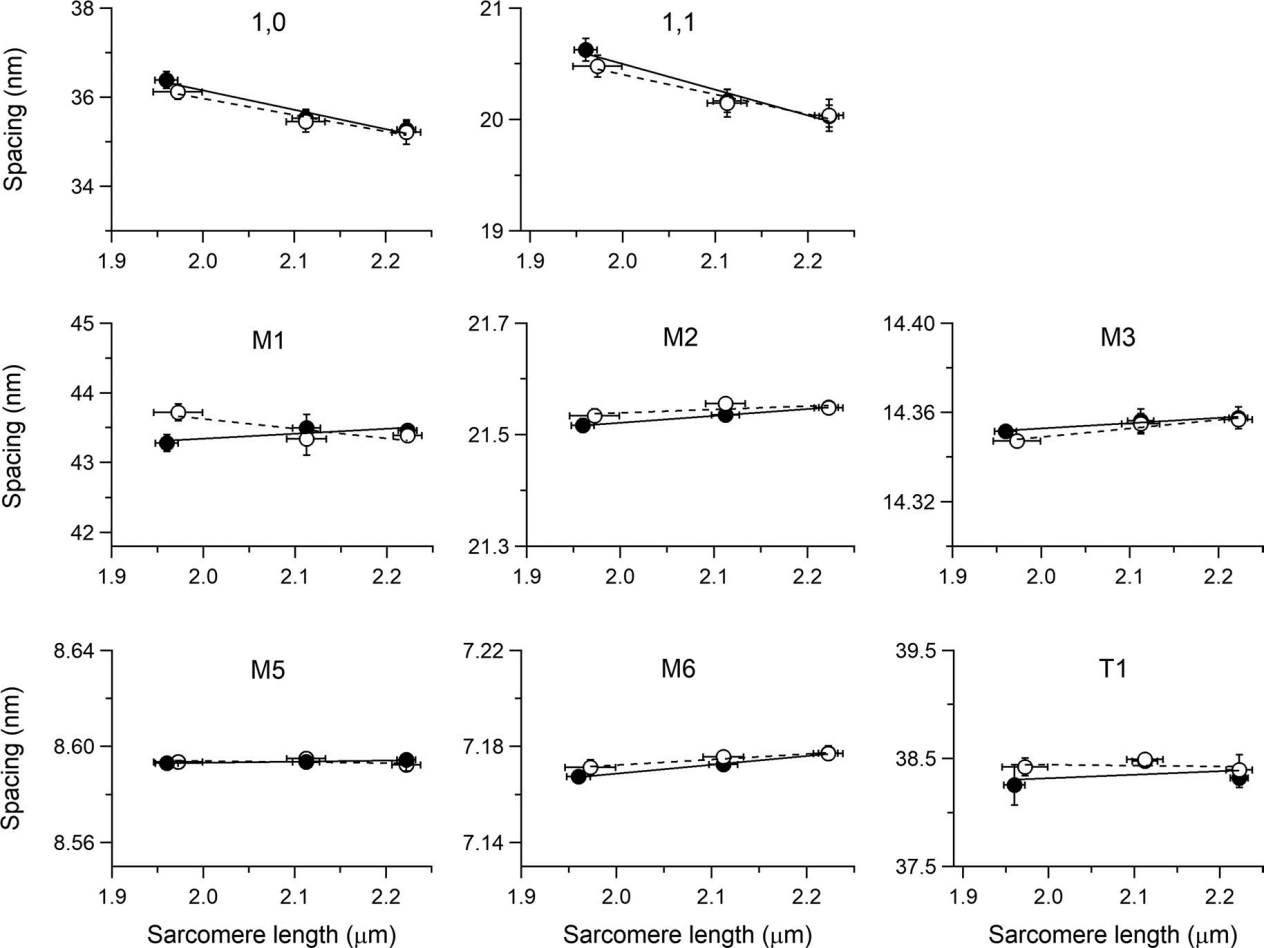
**SL dependence of the spacing of I_1,0_ and I_1,1_, the myosin-based M1–M6 meridional reflections, and the troponin-based T1 reflection.** Filled circles, control; open circles, 10^−7^ M ISO. Continuous and dashed lines are the linear fit to filled and open circles, respectively. Mean ± SEM, four trabeculae.

**Figure 6.**
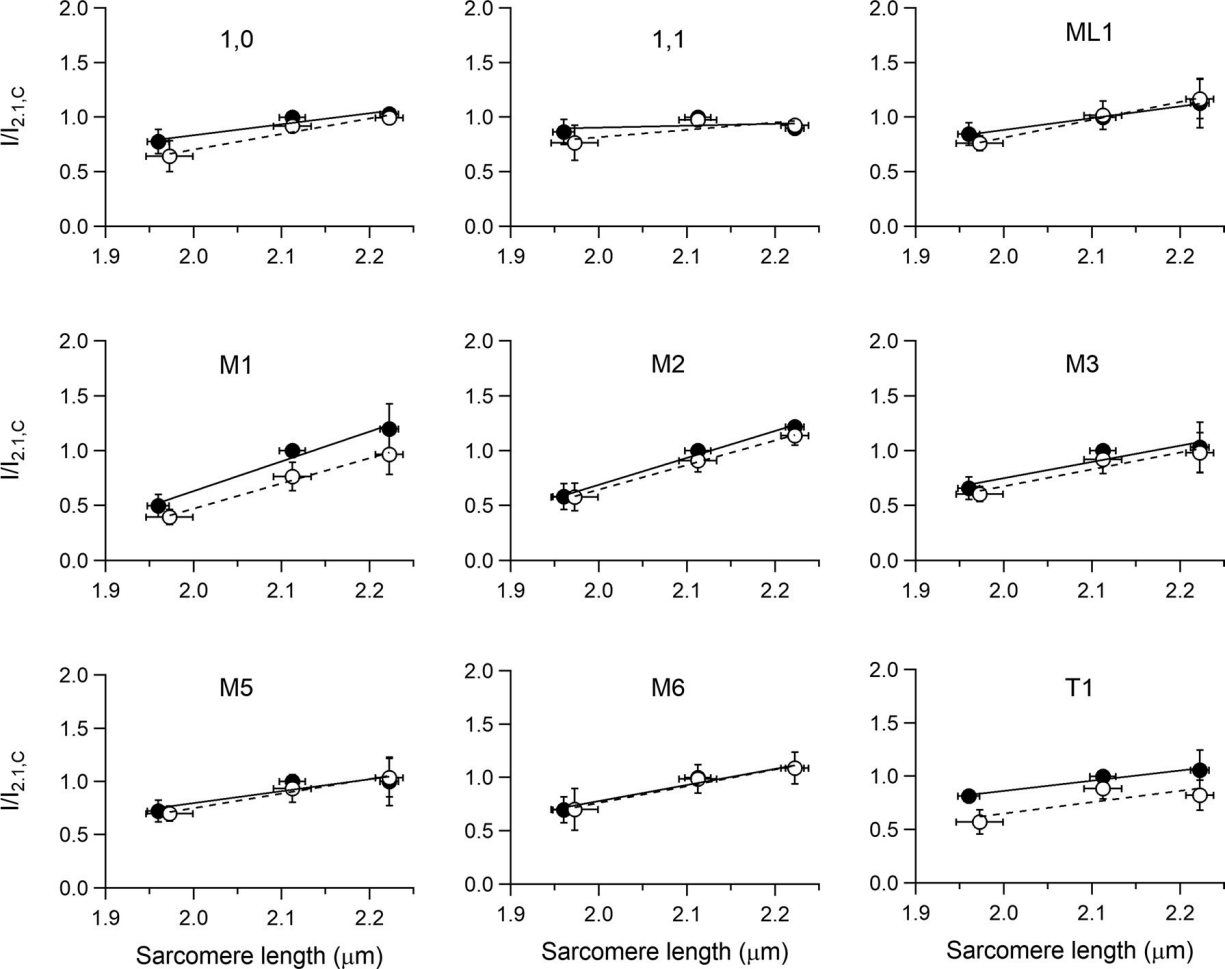
**SL dependence of the intensities of the 1,0 and 1,1, the myosin-based ML1 layer line and M1–M6 meridional, and the troponin-based T1 reflections.** Intensities are relative to the intensity in control at SL 2.11 µm. Data, symbols, and lines as explained in Fig 5.

The intensity distributions of the low-angle equatorial reflections 1,0 and 1,1, obtained by integrating the 2-D pattern across the equator, are shown in Fig. 4 A. Superimposed distributions refer to patterns collected in diastole at SL 1.96 µm (magenta), 2.11 µm (black), and 2.22 µm (green). At the longer SL, both reflections are farther from the center of the pattern, indicating compression of the hexagonal lattice formed by thick and thin filaments (Matsubara and Elliott, 1972; Haselgrove and Huxley, 1973; de Tombe et al., 2010). The SL dependence of the spacing of the equatorial reflections is shown in the graphs of the first row of Fig. 5, filled circles: the spacing of the 1,0 reflection, measuring the distance between lattice planes formed by the thick filament (*d*_1,0_), is 35.5 nm at SL 2.11 µm and reduces by 2.3% from 1.96 to 2.22 µm. From the inspection of the intensity distributions in Fig. 4 A, the peaks of the equatorial reflections appear smaller at shorter SL (especially at 1.96 µm). Indeed, the integrated intensities of the 1,0 ( I_1,0_) and 1,1 (I_1,1_) reflections increase by 33% and 4%, respectively, going from SL 1.96 µm to 2.22 µm (top left and top center panels in Fig. 6, filled circles).

Also, the axial intensity distribution of the ML1 (Fig. 4 B), from the ordered helical disposition of the myosin molecules, shows a reduced maximum with the reduction in SL. The same behavior in relation to SL is shown by all the myosin-based meridional reflections (Fig. 4 C): the M3, from the axial repeat of the myosin heads emerging in crowns of three pairs every 14.3 nm; the M6, mainly related to a structure in the filament backbone with the same periodicity of the M3 (Reconditi et al., 2004; Huxley et al., 2006); and the so called “forbidden” reflections (M1, M2, M4, and M5), associated with the perturbation of the axial repeat of three consecutive crowns within each 43-nm repeat in the C zone (Malinchik and Lednev, 1992; Reconditi et al., 2014). Among them, the M1 also has the contribution of the MyBP-C, present in the C zone of the thick filament with a ∼43-nm periodicity (Rome et al., 1973; Luther et al., 2011), and has, on its high-angle side, the peak of the actin-based reflection due to the 38-nm axial repeat of troponin (T1). Apart from the intensity, the fine structure (Fig. 4 C) and spacing of all the meridional reflections (graphs in the middle and bottom rows of Fig. 5, filled circles) are roughly identical at all SLs in the range studied. The position of the centroid of the myosin-based reflections (middle and bottom rows of Fig. 5, filled circles) shows a minimal increase with SL: the spacing of the M6 reflection (S_M6_, 7.173 nm at 2.11 µm) increases by ∼0.13% from SL 1.96 to 2.22 µm while the spacing of the M5 (S_M5_, 8.594 nm at 2.11 µm) and that of the M3 (S_M3_, 14.356 nm at 2.11 µm) increase by only ∼0.02% and 0.04%, respectively.

The M3 intensity profile due to x-ray interference between the two halves of the thick filament (Linari et al., 2000) shows one main peak with a small satellite on the high-angle side (Fig. 4 C, arrow; see also Fig. 8 E), typical of the OFF state of the myosin heads. Here the heads lie on the surface of the thick filament folded back toward the center of the sarcomere (Zoghbi et al., 2008; Reconditi et al., 2011, 2017; Linari et al., 2015), and this configuration does not appear to be affected by the increase in SL. The other parameters marking the resting state of the thick filament are the intensity of the ML1 (I_ML1_) and the intensity of the myosin-based meridional reflections (I_M1_, I_M2_, I_M3_, I_M5_, and I_M6_). All these reflections show a monotonic increase of intensity with the increase in SL from 1.96 to 2.22 µm (filled circles in Fig. 6), which spans from doubling (I_M1_ and I_M2_) to an ∼50% increase (I_M3_ and I_M6_) to ∼30% increase (I_ML1_ and I_M5_). Also notably, the intensity of T1 (I_T1_) increases by ∼30%.

**Figure 7.**
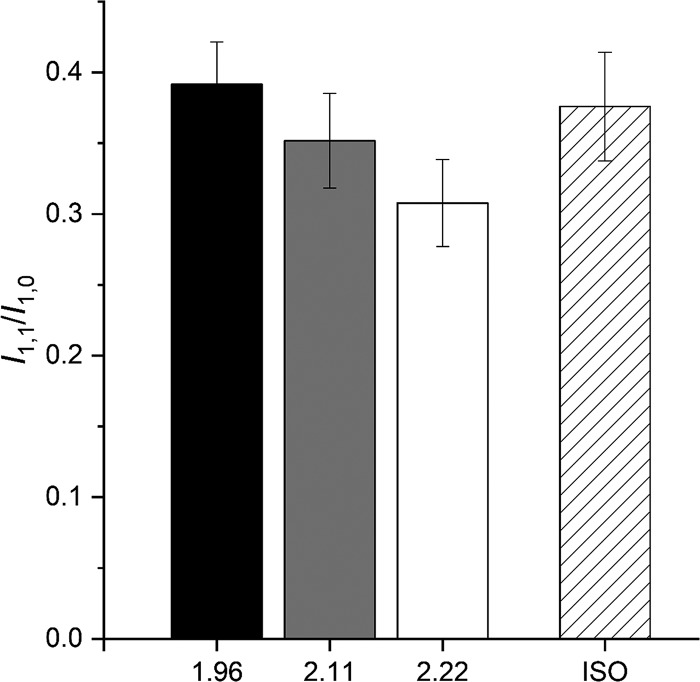
**Intensity ratio of I_1,1_ over I_1,0_ (I_1,1_/I_1,0_) during the diastole at different SL.** Black, 1.96 µm; gray, 2.11 µm; white, 2.22 µm. Dashed, 10^−7^ M ISO at SL 2.11 µm. Means ± SEM, four trabeculae.

I_1,0_ increases with SL more than I_1,1_. Consequently, as shown in Fig. 7, the intensity ratio, I_1,1_/I_1.0_, reduces with the increase in SL, from 0.39 at SL 1.96 µm (black) to 0.35 at 2.11 µm (gray) and 0.31 at 2.22 µm (white), in accordance with the finding reported by Ait-Mou et al. (2016) for the same preparation.

### Effects of the addition of ISO on the x-ray pattern

**Figure 8.**
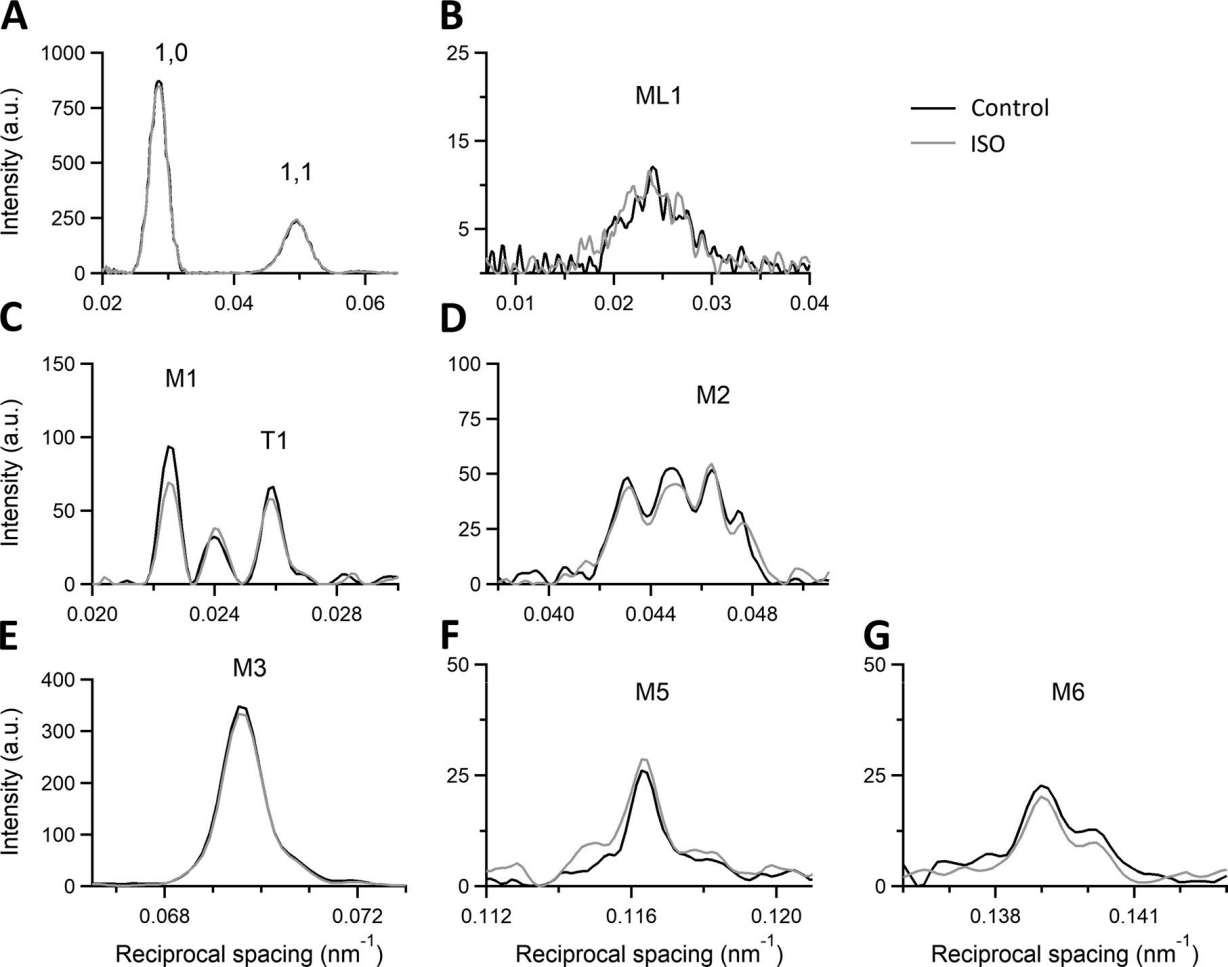
**Effect of ISO on the 1-D intensity distribution from x-ray diffraction patterns during the diastole.** SL 2.11 µm; black (same trace as black in Fig. 4 ) control; gray, in the presence of 10^−7^ M ISO. **(A)** Intensity distribution along the equatorial axis with indicated low-angle I_1,0_ and I_1,1_. **(B)** Intensity distribution along the meridional direction of ML1. **(C–G)** Meridional intensity distribution of the analyzed myosin-based reflections from M1 to M6 and of the troponin-based T1 reflection. Camera length 1.6 m, total exposure time for each condition, 120 ms, summed from four trabeculae.

The addition of the β-adrenergic effector ISO 10^−7^ M to the perfusion solution with [Ca^2+^]_o_ 1 mM at SL 2.11 µm potentiates *T*_P_ to 1.71 *T*_P,C_ (Fig. 1 B and Table 1). The corresponding effects on the x-ray diffraction pattern of the trabecula in diastole are shown in Fig. 8, where the intensity distributions of the equatorial reflections (Fig. 8 A), the ML1 (Fig. 8 B), and the meridional reflections M1/T1 (Fig. 8 C), M2 (Fig. 8 D), M3 (Fig. 8 E), M5 (Fig. 8 F), and M6 (Fig. 8 G) in ISO (gray) are superimposed on those in control (black). It is evident that, apart from a small reduction in the peak intensity of the low-angle peak of M1, none of the parameters for all reflections, including the fine structure, are affected by the addition of ISO. X-ray data in diastole with ISO have been collected at all three SLs used for the preceding test on the SL dependence of the pattern. The relations versus SL of both spacing (Fig. 5) and intensity (Fig. 6) of the various reflections in ISO (open circles) are quite similar to those determined in control (filled circles), with the only exceptions of I_M1_ (middle left panel of Fig. 6) and I_T1_ (bottom right panel of Fig. 6), which exhibit a small but systematic reduction ∼20%) so that, as shown by the vertical shift of the respective linear fits, the ISO relations (dashed lines) lie below those in control (continuous line). As observed in control, also in ISO, I_1,0_ increases with SL (+55% from SL 1.98 to 2.22 µm) more than the I_1,1_ (+21%; top left and top center panels in Fig. 6, open circles), so that for the same SL, as shown at SL 2.11 µm in Fig. 7, the ratio I_1,1_/I_1,0_ in ISO (dashed) is not significantly different from that in control (gray).

## Discussion

### Inotropic interventions do not affect the OFF state of the thick filament in diastole

This paper reports an x-ray diffraction study of the effects on the regulatory state of the thick filament of electrically paced intact trabeculae from rat heart in diastole of two inotropic interventions that almost double the *T*_P_. The first intervention is to increase SL in the range 1.95–2.22 µm, to exploit the LDA that is the cellular basis of the Frank–Starling law (de Tombe et al., 2010); the second is the addition to the perfusion solution of the β-adrenergic effector ISO 10^−7^ M that promotes the PKA-dependent increase in the degree of phosphorylation of many sarcomeric proteins, including MyBP-C. This thick filament accessory protein in demembranated preparations is thought to potentiate the contraction, in relation to its degree of phosphorylation, via the disruption of the IHM and mobilization of the myosin heads from their OFF state even at low [Ca^2+^] (Colson et al., 2012; Kensler et al., 2017).

The results of our experiments do not support the view that these inotropic interventions act through the mobilization of myosin heads before and independently of the Ca^2+^-dependent thin filament activation, as all the x-ray signals marking the OFF state of the thick filament are preserved independent of the increase in SL or addition of ISO. In particular, mobilization of myosin heads from their OFF state is contradicted by the behavior of the following parameters: (a) the intensity of ML1, which depends on the ordered disposition of the myosin heads along three-stranded helical tracks on the surface of the thick filament; (b) the intensity and fine structure of M3, which depend on the OFF state of the myosin heads lying on the surface of the thick filament folded back toward the center of the sarcomere; (c) the intensity of the forbidden reflections (M2, M5), which depends on the perturbation in triplets of the axial repeat of myosin heads in the C zone; and (d) the intensity ratio I_1,1_/I_1,0_, which depends on the proximity of head mass to the thick filament.

In contrast to these results, myosin head mobilization away from their resting state has been consistently found in demembranated cardiac myocytes at low [Ca^2+^] following whatever inotropic intervention, either increase in SL (Kampourakis et al., 2016; Zhang et al., 2017), increase in MyBP-C phosphorylation (Colson et al., 2012), or increase in RLC phosphorylation (Colson et al., 2010; Kampourakis et al., 2016). Notably, the results do not depend on the structural method adopted, either fluorescent probes in the RLC (Kampourakis et al., 2016; Zhang et al., 2017), or x-ray diffraction (Colson et al., 2010, 2012). Moreover, the head mobilization by MyBP-C phosphorylation has been recently confirmed by EM on isolated thick filaments (Kensler et al., 2017). Under these conditions, the only explanation for the different sensitivity to inotropic interventions should be related to the preparation itself. It is possible that in permeabilized myocytes (and in isolated thick filaments), the IHM interactions are weakened by the manipulation, so that any inotropic intervention that implies further weakening of the intra- and intermolecular interactions responsible for the IHM state is sufficient to disrupt the state and release the heads away from the proximity of the thick filament backbone. Actually, in permeabilized myocytes at [Ca^2+^] = 10^−9^ M (pCa 9), myosin heads can already be substantially away from their OFF state, as demonstrated with the fluorescent probe in the RLC (Kampourakis et al., 2016). In this respect, it is worth noting that in skinned fibers from mammalian skeletal muscle, x-ray meridional reflections are weaker, the fine structure of M3 reflection is different from that in the intact preparation, and the whole x-ray pattern partially recovers that of the resting intact muscle with the osmotic recovery of the lattice dimension (Caremani et al., 2017).

### An explanation for the SL-dependent increase of the intensity of all the reflections

A general effect of the increase in SL from 1.96 to 2.22 µm is the increase in the intensity of all the reflections mentioned above, and also of the actin-based meridional T1 reflection (Fig. 6). This finding, unexpected on the basis of similar measurements done in the resting fiber from frog skeletal muscle (Reconditi et al., 2014), likely depends on some basic structural differences between the two striated muscles. As reported in the literature, (a) the length of the thin filament (*L*_A_) is 0.1 µm longer in cardiac muscle (1.04 µm) than in frog skeletal muscle (0.94 µm; Burgoyne et al., 2008), and (b) the width of the Z line (Z_w_) is ∼50 nm larger in the cardiac muscle (100 nm) than in the frog skeletal muscle (Luther, 2009). The minimum SL at which there is no double overlap of the thin filament at the center of the sarcomere (SL_min_) for either muscle can be calculated with the equation SL_min_ = Z_w_ + 2 × *L*_A_ and is 2.18 µm for the cardiac muscle and 1.93 µm for the skeletal muscle. It is evident, from these structural considerations, that in the trabecula for SLs < 2.18 µm, and thus for a large portion of the SL range explored by x-rays in this study (1.96–2.22 µm), there is a small but increasing portion of thin filament undergoing double overlap as SL decreases. This is likely the structural basis for the perturbation of the ordered 3-D disposition of the filaments in a progressive way with the reduction of SL below 2.18 µm.

Another result that is likely related to thin filament structure is the decrease of the intensity ratio of equatorial reflections (I_1,1_/I_1,0_) with the increase in SL. As shown by the top left and top center panels in Fig. 6, the reduction is mainly related to the smaller SL-dependent increase of I_1,1_ with respect to I_1,0_. Notably, a reduction of I_1,1_ with an increase in SL, without reduction in I_1,0_, has been reported for relaxed skeletal muscle (Millman, 1998 and references therein) and attributed to the increase of lateral thin filament disorder with the increase in SL and the corresponding decrease of the length of the thin filament overlapping the thick filament, and thus constrained within the double hexagonal lattice. In the heart muscle, due to the larger thin filament length, the increase in SL in the range explored by x-ray (1.95–2.22 µm) has the combined effect of reducing the double thin filament overlap at the sarcomere center and increasing the not overlapped portion of thin filament in the I band, in this way explaining the reduced increase of I_1,1_ and consequently the reduction in the ratio I_1,1_/I_1,0_. This analysis demonstrates that, even if the change in the ratio (see also Ait-Mou et al., 2016) with SL is in the opposite direction of that expected by a radial shift of the mass of the myosin heads moving away from the helical track, there are other structural reasons against the usual interpretation of the length-dependent changes in I_1,1_/I_1,0_ in terms of radial movement of the mass.

The OFF state of the thick filament is also characterized by short backbone periodicity, as measured by the spacing of the myosin-based meridional reflections, in particular the M6 reflection (S_M6_ = 7.17 nm), which is the second order of a periodic structure with the same periodicity of the myosin heads at rest (14.35 nm; Huxley et al., 2006; Reconditi et al., 2017), but mainly originating from the backbone of the thick filament. M6, with respect to M3, is much less sensitive to head movements that may influence its spacing, and thus is a better measure of the change in extension of the filament (either elastic or structural; Reconditi et al., 2004; Brunello et al., 2014). Actually, as shown in the middle and bottom rows of Fig. 5, the spacing of all the myosin-based meridional reflections slightly increases with SL. In particular, the increase of S_M6_ at SL 2.22 µm (+0.13%) is more than twice that of S_M3_. Notably, 2.22 µm is the SL at which the passive tension starts to rise (green diamond in Fig. 1). In this respect, the behavior of the spacing of M6 and M3 reflections is quite similar to that in frog skeletal muscle fibers at SL ∼2.7 µm, which in this preparation is the SL at which the passive force starts to rise. Also in that case, S_M6_ rise leads S_M3_ rise (Reconditi et al., 2014, open symbols in Fig. 7 D). This common behavior at the threshold of the structural response of the thick filament to the passive force indicates that the stress sensitivity shown through changes in S_M6_ cannot be exclusively related to LDA in the trabecula, but is a common feature of striated muscle.

In conclusion, based on the response to SL increase of equatorial and myosin-based meridional and layer line reflections, the SL-dependent inotropic effect at the basis of LDA does not imply any disruption of the IHM characterizing the OFF state of the myosin motors at low Ca^2+^.

### A specific effect of ISO on the reflections based on MyBP-C and troponin

ISO exerts its positive inotropic action through the PKA-induced phosphorylation of several sarcomeric proteins—that is, (a) proteins involved in the handling of Ca^2+^ (L-type Ca channels, ryanodine receptors, and phospholamban), (b) TnI, the phosphorylation of which induces a faster Ca^2+^ dissociation from troponin C, and (c) MyBP-C, which is believed to exert, also at low Ca^2+^, a phosphorylation-dependent disruption of the IHM state and mobilization of the myosin heads. This multiple action of ISO accounts for its positive inotropic effect on the twitch of the intact trabecula, which is accompanied by a faster rate of force rise and relaxation (positive lusitropic effect; Fig. 1 B).

In this study, we find that in the diastole of an electrically paced intact trabecula, the intensity, fine structure, and spacing of the x-ray reflections and their SL dependence are in general not affected by the addition of ISO 10^−7^ M, which induces a 71% increase in T_P_ .

Two exceptions concern the intensity of the cluster around the M1 reflection, which includes the contribution of the MyBP-C present in the C zone of the thick filament, and the intensity of the T1 reflection, which arises from the troponin periodicity along the thin filament. In ISO (open circles in Fig. 6), both reflections are ∼20% lower than in control (filled circles). The paired *t* test indicates that the differences are significant (P < 0.03).

To interpret these changes in terms of the regulatory state of the filaments, it must be considered that (a) the effect on these two reflections is not present using as inotropic intervention the increase in SL, and (b) the structural change induced by ISO is strictly limited to M1 and T1 and thus, very likely, to MyBP-C on the thick filament and troponin on the thin filament, which are the filament proteins that represent the target of PKA-dependent phosphorylation induced by ISO. However, T1 changes do not necessarily have to be attributed to a direct effect of TnI phosphorylation. The coupled effect of ISO on M1 and T1 reflections could be explained by the dynamic interactions of MyBP-C with the thin filament, which are strengthened by phosphorylation (Moos et al., 1978; Rybakova et al., 2011; Pfuhl and Gautel, 2012; Mun et al., 2014).

### The role of inotropic interventions in thick filament activation

**Figure 9.**
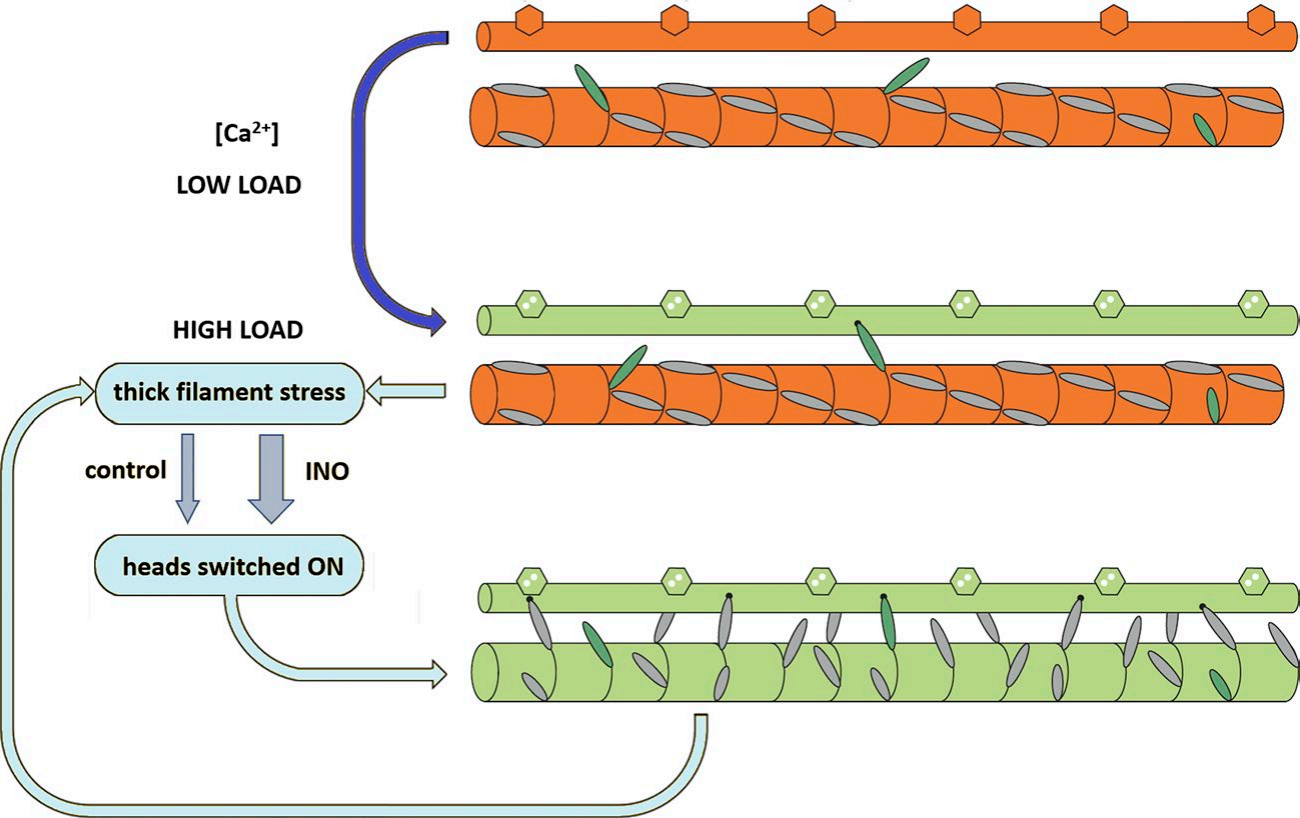
**Dual-filament regulation in cardiac muscle and the effect of inotropic interventions on the thick filament.** The thin filament is drawn with hexagons symbolizing troponin and it is switched ON (orange to green) by calcium binding (blue arrow); the thick filament is switched ON (orange to green) during loaded contraction through a positive feedback between force on the thick filament and recruitment of heads from their OFF state (cyan loop). Inotropic interventions (INO) like increase in SL or addition of ISO to the solution increase the gain of the feedback loop (represented by the width of the gray arrows). On the thick filament, constitutively ON myosin heads are green; myosin heads under thick filament control are gray. Adapted from Linari et al. (2015), Fig. 9 is reprinted with permission from *Nature*.

None of the signals marking the OFF state of the myosin motors during diastole are affected by inotropic interventions that can double the systolic force, such as increased degree of phosphorylation of MyBP-C or LDA. Thus, inotropic effectors present during diastole in relation to either neuro-humoral control of the heart or ventricle filling exert their effect on the thick filament activation only once the Ca^2+^-dependent thin filament activation is ON. The idea that thick filament activation is based on a mechanism downstream with respect to thin filament activation is also supported by the finding that switching ON of the myosin heads is independent of the diastolic SL and depends on the systolic SL (Reconditi et al., 2017). All these data converge toward a unique explanation based on the role of thick filament mechanosensing in striated muscle: recruitment of myosin motors from their energy-saving OFF state depends on the stress on the thick filament (Linari et al., 2015; Reconditi et al., 2017; Piazzesi et al., 2018). How do inotropic interventions operate in this scenario? The evidence that in relaxed skinned myocytes inotropic interventions promote the release of the heads away from the backbone of the thick filament by weakening the interactions responsible for the IHM state of the myosin molecule strongly sustains the idea that both LDA and MyBP-C phosphorylation set the gain of the positive feedback that relates motor recruitment to the stress on the thick filament (Fig. 9). It must be noted that this mechanism, if it is confirmed by direct investigation, is independent of and complementary to that operating on thin filament–based regulation through protein phosphorylation and LDA.

The application of the experimental approach described here to intact trabeculae of mutant mouse models, in which either MyBP-C phosphorylation (Colson et al., 2012) or LDA-modulating titin stiffness (Fukuda et al., 2010) can be manipulated by site-directed mutagenesis, is the next necessary step for testing the above conclusion and defining the communicating pathway and temporal sequence of the dynamic interactions between MyBP-C, titin, and contractile and regulatory proteins.

Mutations in cardiac MyBP-C and in the domains of cardiac myosin that interact with MyBP-C are responsible for 80% of cases of hypertrophic cardiomyopathy (HCM), an inherited heart disease characterized by thickening of the ventricular wall that diminishes the relaxation capacity and ventricular filling. These HCM-causing mutations are thought to disrupt the intermolecular interactions controlling the formation of the IHM and produce the hypercontractility that leads to HCM (Spudich, 2015; Alamo et al., 2017; Trivedi et al., 2018). The conclusion in this paper that the stress-sensing mechanism that switches ON myosin motors is modulated by the inotropic interventions opens a new scenario in which the HCM-causing mutations would operate by lowering the force threshold of the switch that controls the thick filament activation.

## References

[bib1] Ait-Mou Y., Hsu K., Farman G.P., Kumar M., Greaser M.L., Irving T.C., and de Tombe P.P. 2016 Titin strain contributes to the Frank-Starling law of the heart by structural rearrangements of both thin- and thick-filament proteins. Proc. Natl. Acad. Sci. USA. 113:2306–2311. 10.1073/pnas.1516732113 26858417 PMC4776536

[bib2] Alamo L., Wriggers W., Pinto A., Bártoli F., Salazar L., Zhao F.-Q., Craig R., and Padrón R. 2008 Three-dimensional reconstruction of tarantula myosin filaments suggests how phosphorylation may regulate myosin activity. J. Mol. Biol. 384:780–797. 10.1016/j.jmb.2008.10.013 18951904 PMC2729561

[bib3] Alamo L., Ware J.S., Pinto A., Gillilan R.E., Seidman J.G., Seidman C.E., and Padrón R. 2017 Effects of myosin variants on interacting-heads motif explain distinct hypertrophic and dilated cardiomyopathy phenotypes. eLife. 6:e24634 10.7554/eLife.24634 28606303 PMC5469618

[bib4] Allen D.G., and Kentish J.C. 1985 The cellular basis of the length-tension relation in cardiac muscle. J. Mol. Cell. Cardiol. 17:821–840. 10.1016/S0022-2828(85)80097-3 3900426

[bib5] Brunello E., Caremani M., Melli L., Linari M., Fernandez-Martinez M., Narayanan T., Irving M., Piazzesi G., Lombardi V., and Reconditi M. 2014 The contributions of filaments and cross-bridges to sarcomere compliance in skeletal muscle. J. Physiol. 592:3881–3899. 10.1113/jphysiol.2014.276196 25015916 PMC4192709

[bib6] Burgoyne T., Muhamad F., and Luther P.K. 2008 Visualization of cardiac muscle thin filaments and measurement of their lengths by electron tomography. Cardiovasc. Res. 77:707–712. 10.1093/cvr/cvm117 18178575 PMC5436745

[bib7] Caremani M., Pinzauti F., Reconditi M., Piazzesi G., Stienen G.J.M., Lombardi V., and Linari M. 2016 Size and speed of the working stroke of cardiac myosin in situ. Proc. Natl. Acad. Sci. USA. 113:3675–3680. 10.1073/pnas.1525057113 26984499 PMC4822625

[bib8] Caremani M., Fusi L., Reconditi M., Piazzesi G., Narayanan T., Irving M., Lombardi V., and Brunello E. 2017 Structural changes in the thick filaments during activation of demembranated skeletal muscle fibers. Biophys. J. 112:181a 10.1016/j.bpj.2016.11.1004

[bib9] Cazorla O., Wu Y., Irving T.C., and Granzier H. 2001 Titin-based modulation of calcium sensitivity of active tension in mouse skinned cardiac myocytes. Circ. Res. 88:1028–1035. 10.1161/hh1001.090876 11375272

[bib10] Colson B.A., Locher M.R., Bekyarova T., Patel J.R., Fitzsimons D.P., Irving T.C., and Moss R.L. 2010 Differential roles of regulatory light chain and myosin binding protein-C phosphorylations in the modulation of cardiac force development. J. Physiol. 588:981–993. 10.1113/jphysiol.2009.183897 20123786 PMC2849963

[bib11] Colson B.A., Patel J.R., Chen P.P., Bekyarova T., Abdalla M.I., Tong C.W., Fitzsimons D.P., Irving T.C., and Moss R.L. 2012 Myosin binding protein-C phosphorylation is the principal mediator of protein kinase A effects on thick filament structure in myocardium. J. Mol. Cell. Cardiol. 53:609–616. 10.1016/j.yjmcc.2012.07.012 22850286 PMC3472100

[bib12] de Tombe P.P., Mateja R.D., Tachampa K., Ait Mou Y., Farman G.P., and Irving T.C. 2010 Myofilament length dependent activation. J. Mol. Cell. Cardiol. 48:851–858. 10.1016/j.yjmcc.2009.12.017 20053351 PMC2854194

[bib13] Ebashi S., Endo M., and Otsuki I. 1969 Control of muscle contraction. Q. Rev. Biophys. 2:351–384. 10.1017/S0033583500001190 4935801

[bib14] Farman G.P., Gore D., Allen E., Schoenfelt K., Irving T.C., and de Tombe P.P. 2011 Myosin head orientation: a structural determinant for the Frank-Starling relationship. Am. J. Physiol. Heart Circ. Physiol. 300:H2155–H2160. 10.1152/ajpheart.01221.2010 21460195 PMC3119094

[bib15] Fukuda N., Terui T., Ishiwata S., and Kurihara S. 2010 Titin-based regulations of diastolic and systolic functions of mammalian cardiac muscle. J. Mol. Cell. Cardiol. 48:876–881. 10.1016/j.yjmcc.2009.11.013 19962382

[bib16] Gordon A.M., Homsher E., and Regnier M. 2000 Regulation of contraction in striated muscle. Physiol. Rev. 80:853–924. 10.1152/physrev.2000.80.2.853 10747208

[bib17] Harris S.P., Bartley C.R., Hacker T.A., McDonald K.S., Douglas P.S., Greaser M.L., Powers P.A., and Moss R.L. 2002 Hypertrophic cardiomyopathy in cardiac myosin binding protein-C knockout mice. Circ. Res. 90:594–601. 10.1161/01.RES.0000012222.70819.64 11909824

[bib18] Haselgrove J.C., and Huxley H.E. 1973 X-ray evidence for radial cross-bridge movement and for the sliding filament model in actively contracting skeletal muscle. J. Mol. Biol. 77:549–568. 10.1016/0022-2836(73)90222-2 4541885

[bib19] Herron T.J., Korte F.S., and McDonald K.S. 2001 Power output is increased after phosphorylation of myofibrillar proteins in rat skinned cardiac myocytes. Circ. Res. 89:1184–1190. 10.1161/hh2401.101908 11739284

[bib20] Hidalgo C., and Granzier H. 2013 Tuning the molecular giant titin through phosphorylation: role in health and disease. Trends Cardiovasc. Med. 23:165–171. 10.1016/j.tcm.2012.10.005 23295080 PMC3622841

[bib21] Huxley H.E. 1973 Structural Changes in the Actin- and Myosin-containingc Filaments during Contraction. Cold Spring Harb. Symp. Quant. Biol. 37:361–376. 10.1101/SQB.1973.037.01.046

[bib22] Huxley A.F., Lombardi V., and Peachey L.D. 1981 A system for fast recording of longitudinal displacement of a striated muscle fibre. J. Physiol. 317:12P–13P.

[bib23] Huxley H., Reconditi M., Stewart A., and Irving T. 2006 X-ray interference studies of crossbridge action in muscle contraction: evidence from quick releases. J. Mol. Biol. 363:743–761. 10.1016/j.jmb.2006.08.075 17007871

[bib24] Kampourakis T., Yan Z., Gautel M., Sun Y.-B., and Irving M. 2014 Myosin binding protein-C activates thin filaments and inhibits thick filaments in heart muscle cells. Proc. Natl. Acad. Sci. USA. 111:18763–18768. 10.1073/pnas.1413922112 25512492 PMC4284530

[bib25] Kampourakis T., Sun Y.B., and Irving M. 2016 Myosin light chain phosphorylation enhances contraction of heart muscle via structural changes in both thick and thin filaments. Proc. Natl. Acad. Sci. USA. 113:E3039–E3047. 10.1073/pnas.1602776113 27162358 PMC4889392

[bib26] Kensler R.W., Craig R., and Moss R.L. 2017 Phosphorylation of cardiac myosin binding protein C releases myosin heads from the surface of cardiac thick filaments. Proc. Natl. Acad. Sci. USA. 114:E1355–E1364. 10.1073/pnas.1614020114 28167762 PMC5338423

[bib27] Korte F.S., McDonald K.S., Harris S.P., and Moss R.L. 2003 Loaded shortening, power output, and rate of force redevelopment are increased with knockout of cardiac myosin binding protein-C. Circ. Res. 93:752–758. 10.1161/01.RES.0000096363.85588.9A 14500336

[bib28] Kumar M., Govindan S., Zhang M., Khairallah R.J., Martin J.L., Sadayappan S., and de Tombe P.P. 2015 Cardiac Myosin-binding Protein C and Troponin-I Phosphorylation Independently Modulate Myofilament Length-dependent Activation. J. Biol. Chem. 290:29241–29249. 10.1074/jbc.M115.686790 26453301 PMC4705930

[bib29] Labeit S., Gautel M., Lakey A., and Trinick J. 1992 Towards a molecular understanding of titin. EMBO J. 11:1711–1716. 10.1002/j.1460-2075.1992.tb05222.x 1582406 PMC556628

[bib30] Linari M., Piazzesi G., Dobbie I., Koubassova N., Reconditi M., Narayanan T., Diat O., Irving M., and Lombardi V. 2000 Interference fine structure and sarcomere length dependence of the axial x-ray pattern from active single muscle fibers. Proc. Natl. Acad. Sci. USA. 97:7226–7231. 10.1073/pnas.97.13.7226 10860988 PMC16527

[bib31] Linari M., Brunello E., Reconditi M., Fusi L., Caremani M., Narayanan T., Piazzesi G., Lombardi V., and Irving M. 2015 Force generation by skeletal muscle is controlled by mechanosensing in myosin filaments. Nature. 528:276–279. 10.1038/nature15727 26560032

[bib32] Lombardi V., and Piazzesi G. 1990 The contractile response during steady lengthening of stimulated frog muscle fibres. J. Physiol. 431:141–171. 10.1113/jphysiol.1990.sp018324 2100305 PMC1181768

[bib33] Luther P.K. 2009 The vertebrate muscle Z-disc: sarcomere anchor for structure and signalling. J. Muscle Res. Cell Motil. 30:171–185. 10.1007/s10974-009-9189-6 19830582 PMC2799012

[bib34] Luther P.K., Winkler H., Taylor K., Zoghbi M.E., Craig R., Padrón R., Squire J.M., and Liu J. 2011 Direct visualization of myosin-binding protein C bridging myosin and actin filaments in intact muscle. Proc. Natl. Acad. Sci. USA. 108:11423–11428. 10.1073/pnas.1103216108 21705660 PMC3136262

[bib35] Malinchik S.B., and Lednev V.V. 1992 Interpretation of the X-ray diffraction pattern from relaxed skeletal muscle and modelling of the thick filament structure. J. Muscle Res. Cell Motil. 13:406–419. 10.1007/BF01738036 1401037

[bib36] Mamidi R., Gresham K.S., and Stelzer J.E. 2014 Length-dependent changes in contractile dynamics are blunted due to cardiac myosin binding protein-C ablation. Front. Physiol. 5:461 10.3389/fphys.2014.00461 25520665 PMC4251301

[bib37] Mamidi R., Gresham K.S., Verma S., and Stelzer J.E. 2016 Cardiac Myosin Binding Protein-C Phosphorylation Modulates Myofilament Length-Dependent Activation. Front. Physiol. 7:38 10.3389/fphys.2016.00038 26913007 PMC4753332

[bib38] Matsubara I., and Elliott G.F. 1972 X-ray diffraction studies on skinned single fibres of frog skeletal muscle. J. Mol. Biol. 72:657–669. 10.1016/0022-2836(72)90183-0 4540801

[bib39] Millman B.M. 1998 The filament lattice of striated muscle. Physiol. Rev. 78:359–391. 10.1152/physrev.1998.78.2.359 9562033

[bib40] Moos C., Mason C.M., Besterman J.M., Feng I.-N.M., and Dubin J.H. 1978 The binding of skeletal muscle C-protein to F-actin, and its relation to the interaction of actin with myosin subfragment-1. J. Mol. Biol. 124:571–586. 10.1016/0022-2836(78)90172-9 152359

[bib41] Mun J.Y., Previs M.J., Yu H.Y., Gulick J., Tobacman L.S., Beck Previs S., Robbins J., Warshaw D.M., and Craig R. 2014 Myosin-binding protein C displaces tropomyosin to activate cardiac thin filaments and governs their speed by an independent mechanism. Proc. Natl. Acad. Sci. USA. 111:2170–2175. 10.1073/pnas.1316001111 24477690 PMC3926057

[bib42] Narayanan T., Sztucki M., van Vaerenbergh P., Léonardon J., Gorini J., Claustre L., Sever F., Morse J., and Boesecke P. 2018 A multipurpose instrument for time-resolved ultra-small-angle and coherent X-ray scattering. J. Appl. Cryst. 51 10.1107/S1600576718012748 PMC6276275 30546286

[bib43] Pfuhl M., and Gautel M. 2012 Structure, interactions and function of the N-terminus of cardiac myosin binding protein C (MyBP-C): who does what, with what, and to whom? J. Muscle Res. Cell Motil. 33:83–94. 10.1007/s10974-012-9291-z 22527637

[bib44] Piazzesi G., Caremani M., Linari M., Reconditi M., and Lombardi V. 2018 Thick Filament Mechano-Sensing in Skeletal and Cardiac Muscles: A Common Mechanism Able to Adapt the Energetic Cost of the Contraction to the Task. Front. Physiol. 9:736 10.3389/fphys.2018.00736 29962967 PMC6010558

[bib45] Pinzauti F., Pertici I., Reconditi M., Narayanan T., Stienen G.J.M., Piazzesi G., Lombardi V., Linari M., and Caremani M. 2018 The force and stiffness of myosin motors in the isometric twitch of a cardiac trabecula and the effect of the extracellular calcium concentration. J. Physiol. 596:2581–2596. 10.1113/JP275579 29714038 PMC6023834

[bib46] Reconditi M., Linari M., Lucii L., Stewart A., Sun Y.-B., Boesecke P., Narayanan T., Fischetti R.F., Irving T., Piazzesi G., 2004 The myosin motor in muscle generates a smaller and slower working stroke at higher load. Nature. 428:578–581. 10.1038/nature02380 15058307

[bib47] Reconditi M., Brunello E., Linari M., Bianco P., Narayanan T., Panine P., Piazzesi G., Lombardi V., and Irving M. 2011 Motion of myosin head domains during activation and force development in skeletal muscle. Proc. Natl. Acad. Sci. USA. 108:7236–7240. 10.1073/pnas.1018330108 21482782 PMC3084075

[bib48] Reconditi M., Brunello E., Fusi L., Linari M., Martinez M.F., Lombardi V., Irving M., and Piazzesi G. 2014 Sarcomere-length dependence of myosin filament structure in skeletal muscle fibres of the frog. J. Physiol. 592:1119–1137. 10.1113/jphysiol.2013.267849 24344169 PMC3948567

[bib49] Reconditi M., Caremani M., Pinzauti F., Powers J.D., Narayanan T., Stienen G.J.M., Linari M., Lombardi V., and Piazzesi G. 2017 Myosin filament activation in the heart is tuned to the mechanical task. Proc. Natl. Acad. Sci. USA. 114:3240–3245. 10.1073/pnas.1619484114 28265101 PMC5373356

[bib50] Rome E., Offer G., and Pepe F.A. 1973 X-ray diffraction of muscle labelled with antibody to C-protein. Nat. New Biol. 244:152–154. 10.1038/newbio244152a0 4516378

[bib51] Rybakova I.N., Greaser M.L., and Moss R.L. 2011 Myosin binding protein C interaction with actin: characterization and mapping of the binding site. J. Biol. Chem. 286:2008–2016 https://doi.org/. 10.1074/jbc.M110.170605 21071444 PMC3023497

[bib52] Sagawa K., Maughan W.L., Suga H., and Sunagawa K. 1988 Cardiac Contraction and the Pressure Volume Relationship. Oxford University Press, New York.

[bib53] Spudich J.A. 2014 Hypertrophic and dilated cardiomyopathy: four decades of basic research on muscle lead to potential therapeutic approaches to these devastating genetic diseases. Biophys. J. 106:1236–1249. 10.1016/j.bpj.2014.02.011 24655499 PMC3985504

[bib54] Spudich J.A. 2015 The myosin mesa and a possible unifying hypothesis for the molecular basis of human hypertrophic cardiomyopathy. Biochem. Soc. Trans. 43:64–72. 10.1042/BST20140324 25619247 PMC4349527

[bib55] Stewart M.A., Franks-Skiba K., Chen S., and Cooke R. 2010 Myosin ATP turnover rate is a mechanism involved in thermogenesis in resting skeletal muscle fibers. Proc. Natl. Acad. Sci. USA. 107:430–435. 10.1073/pnas.0909468107 19966283 PMC2806748

[bib56] ter Keurs H.E. 2012 The interaction of Ca2+ with sarcomeric proteins: role in function and dysfunction of the heart. Am. J. Physiol. Heart Circ. Physiol. 302:H38–H50. 10.1152/ajpheart.00219.2011 22021327 PMC3334233

[bib57] Trivedi D.V., Adhikari A.S., Sarkar S.S., Ruppel K.M., and Spudich J.A. 2018 Hypertrophic cardiomyopathy and the myosin mesa: viewing an old disease in a new light. Biophys. Rev. 10:27–48. 10.1007/s12551-017-0274-6 28717924 PMC5803174

[bib58] Woodhead J.L., Zhao F.Q., Craig R., Egelman E.H., Alamo L., and Padrón R. 2005 Atomic model of a myosin filament in the relaxed state. Nature. 436:1195–1199. 10.1038/nature03920 16121187

[bib59] Zhang X., Kampourakis T., Yan Z., Sevrieva I., Irving M., and Sun Y.B. 2017 Distinct contributions of the thin and thick filaments to length-dependent activation in heart muscle. eLife. 6:e24081 10.7554/eLife.24081 28229860 PMC5365314

[bib60] Zoghbi M.E., Woodhead J.L., Moss R.L., and Craig R. 2008 Three-dimensional structure of vertebrate cardiac muscle myosin filaments. Proc. Natl. Acad. Sci. USA. 105:2386–2390. 10.1073/pnas.0708912105 18252826 PMC2268146

